# Prefrontal Asymmetry and Parent-Rated Temperament in Infants

**DOI:** 10.1371/journal.pone.0022694

**Published:** 2011-07-28

**Authors:** Vanessa LoBue, James A. Coan, Cat Thrasher, Judy S. DeLoache

**Affiliations:** 1 Department of Psychology, Rutgers University, Newark, New Jersey, United States of America; 2 Department of Psychology, University of Virginia, Charlottesville, Virginia, United States of America; RAND Corporation, United States of America

## Abstract

Indicators of temperament appear early in infancy and remain relatively stable over time. Despite a great deal of interest in biological indices of temperament, most studies of infant temperament rely on parental reports or behavioral tasks. Thus, the extent to which commonly used temperament measures relate to potential biological indicators of infant temperament is still relatively unknown. The current experiment examines the relationship between a common parental report measure of temperament – the Infant Behavior Questionnaire – Revised (IBQ-R) – and measures of frontal EEG asymmetry in infants. We examined associations between the subscales of the IBQ-R and frontal EEG asymmetry scores recorded during a combined series of neutral attentional and putatively emotional recording conditions in infants between 7 and 9 months of age. We predicted that approach-related subscales of the IBQ-R (e.g., Approach, Soothability) would be related to greater left prefrontal asymmetry, while withdrawal-related subscales (e.g., Distress to Limitations, Fear, Falling Reactivity, Perceptual Sensitivity) would be related to greater right prefrontal asymmetry. In the mid- and lateral-frontal regions, Approach, Distress to Limitations, Fear, Soothability, and Perceptual Sensitivity were generally associated with greater left frontal activation (*rs*≥.23, *ps*<0.05), while only Falling Reactivity was associated with greater right frontal activation (*rs*≤−.44, *ps*<0.05). Results suggest that variability in frontal EEG asymmetry is robustly associated with parental report measures of temperament in infancy.

## Introduction

The first signs of human personality are evident in infant temperament [1], [2], [3]. Infant temperament can be defined as “…individual differences in emotional, motor, and attentional reactivity measured by latency, intensity, and recover of response, and self-regulation processes such as effortful control that modulate reactivity,” (pg. 207, [4]). Such individual differences are relatively stable, biologically mediated manifestations of differing behavioral strategies (i.e., methods of responding to changes in the environment) that impart various advantages during early development [5], [6]. For example, some infants can be characterized as more fearful than others, and behave cautiously in social situations. These infants may later be called shy as toddlers. Other, less fearful infants, are more outgoing, social, and receptive to new stimuli, and may be less prone to shyness as they grow older. Contemporary conceptions of infant temperament emphasize its: (1) biological basis, (2) appearance early in infancy, and (3) stability across time and situations.

A large body of work confirms that temperament measured even early in infancy is relatively stable across time, although test-retest reliabilities from infancy to later in childhood and adolescence are modest [4], [6], [7]. Such early, stable temperamental tendencies are widely-believed to be rooted in heritable physiological dispositions to respond characteristically in given contexts or in response to specific types of stimuli [8], [9]. However, despite the prevailing assumption of strong biological influences, infant temperament research is still largely dependent upon parent-report.

Although temperament questionnaires are valuable for measuring individual differences [10]. For example, Fox et al. [10] suggest that temperament fundamentally reflects *physiological* responses to different sensory stimuli, particularly emotionally valenced stimuli, and, along with Posner and Rothbart [11], suggests that temperament is directly related to executive circuits in the developing prefrontal cortex (PFC).

### Prefrontal Asymmetry, Affective Style, and Infant Temperament

Several researchers have emphasized the role of the prefrontal cortex in infant, child, and even adult temperament and personality [12]. Moreover, an important index of prefrontal associations with temperament and personality has been found in electroencephalographic (EEG) measures of *frontal EEG asymmetry* (e.g., [13], [14], [15]). In this work, frontal EEG asymmetry is often measured as a simple difference score obtained by subtracting left from right (ln[right] - ln[left]) cortical alpha power (8–13 Hz in adults, 6–9 Hz in infants, as described below)—a spectral band associated with neural resting states and, thus, thought to reliably index the inverse of neural activity (e.g., [13], [14], [15]). This difference score provides a single, continuous measure of hemispheric asymmetry over the prefrontal cortex thought to provide an important intermediate link between the social, behavioral, psychological, and cellular mechanisms underlying adult personality and infant temperament (cf., [16], [17]). Specifically, frontal EEG asymmetry has been proposed as 1) an index of tendencies to approach or withdraw when presented with positively and negatively valenced stimuli [12], [14], [18], [19]; and 2) a potential endophenotypic biomarker of affective risk and resiliency in the face of stressful life situations [17], [20]. A large number of studies of frontal EEG asymmetry in both adults [12], [21], [22] and infants [14], [18], [19] suggest that left lateralized prefrontal activity indexes approach-related activity and that right-lateralized prefrontal activity indexes withdrawal-related activity.

Nearly 25 years ago, Fox and Davidson [14] introduced the notion that temperament is also closely related to these approach and withdrawal systems—that in fact these systems guide infant exploratory and inhibitory behaviors from birth. A large and growing body of research now supports this view, in adults as well as in children and infants [10], [20], [23], [24], [25], [26], [27]. In their early work, Davidson and Fox [28] observed that 10-month-old infants produced greater activation in the left than right prefrontal areas when viewing photographs of happy as contrasted with sad faces, presumably because of the reward value of happy as opposed to sad faces. Later, Fox and Davidson [29] observed that newborn infants demonstrated relatively greater left prefrontal activity when presented with sucrose than when presented with water, again in proportion to the reward value of sucrose over water.

More recent research has linked frontal EEG asymmetry in infancy to behavioral measures of temperament in older infants. For example, Fox et al. [10] found that right frontal asymmetry at 9 months was related to behavioral inhibition in infants later in the first two years of life. Similarly, Fox et al. [30] observed that in 49- to 62-month-olds, greater social competence corresponded with relatively greater left prefrontal activity, although social withdrawal corresponded with relatively greater right prefrontal activity.

### Frontal EEG Asymmetry and the Infant Behavior Questionnaire

The primary goal in the current experiment was to examine associations between infant frontal EEG asymmetry and what is perhaps the most widely used parental report measure of infant temperament, the Infant Behavior Questionnaire – Revised (IBQ-R [31]). Given the frequency of its use, it is important to investigate the relationship between parent temperament reports on the IBQ-R and concurrent putative biological measures of temperament. Although past studies have examined associations between the IBQ and prefrontal processes in infants, these studies remain somewhat limited in number [15], [32], [33], and to our knowledge none have reported on the IBQ-R and concurrent measures of frontal EEG asymmetry. For example, Henderson et al. [15] used the IBQ to calculate a Negative Reactivity index (comprised of the IBQ's Fear and Distress to Limitations subscales), and observed that this Negative Reactivity index interacted with frontal EEG asymmetry (both collected at 9 to 14 months of age) to predict subsequent social wariness (at 4 years). More specifically, the association they observed between 9-month negative reactivity and 4-year-old social wariness was substantially greater in infants classified as “right frontal” (having asymmetry scores less than zero), than in those classified as “left frontal.” Although they did not use frontal EEG asymmetry as their measure of prefrontal functioning, de Haan et al., [32] did observe that 7-month-olds rated high on the IBQ subscale of Fear showed a relatively enhanced right prefrontal negative-central event-related component—thought to reflect attention during orienting—when viewing fearful, as opposed to happy, faces. Most recently, Schmidt [33] observed that 9-month-old infants categorized as being stably right-lateralized over the mid-prefrontal cortex (using frontal EEG asymmetry scores) had higher maternal ratings on the IBQ subscale of Fear than infants categorized as either stably left-lateralized or variable (between right and left) over the same prefrontal regions. Again, we are unaware of any researchers who have observed direct, zero-order correlations between parental reports of infant temperament using the IBQ-R and the frontal EEG asymmetry score.

### Infant Temperament and The Capability Model of Frontal EEG Asymmetry

In adult measures of frontal EEG asymmetry and personality, the dominant recording mode is the resting condition, a condition thought to reduce contextual biases for the purpose of capturing a person's asymmetry score. Many infant studies of individual differences in frontal EEG asymmetry have attempted to approximate neutral or resting recording conditions. For example, Henderson et al. [15] recorded three minutes of EEG while infants watched a spinning bingo wheel that focused attention in a context of relative emotional neutrality. Similarly, Schmidt [33] recorded EEG in 9-month-old infants as they attended to a neutral computer screen saver. Recently, Coan et al. [20] have argued that the information available in affectively neutral frontal EEG asymmetry recordings can be enhanced by recording during emotional challenges. Specifically, Coan et al. [20] suggested that recording frontal EEG asymmetry during emotional challenges can increase the proportion of variance in frontal EEG asymmetry attributable to stable individual differences and increase the magnitude and reliability of statistical associations between frontal EEG asymmetry and measures of both temperament [20] and psychopathology [34] obtained by other modalities.

Thus, a secondary goal of the current research is to examine associations between the IBQ and frontal EEG asymmetry obtained during emotionally salient situations. Although the studies described above examined frontal EEG asymmetry during resting or neutral conditions (e.g., [15], [33]), relatively few have examined frontal EEG asymmetries recorded during more emotionally salient conditions. As described above, Davidson and Fox [28] and Fox and Davidson [29] examined frontal EEG asymmetry during varying test conditions nearly 25 years ago, but did not at that time relate frontal EEG asymmetry to parental report measures of temperament. More recently, Santesso, Schmidt, and Trainor [35] measured EEG in 9-month-old infants during emotionally-relevant infant-directed speech. They found that EEG was related to the emotional intensity of the speech, in that EEG responses were greatest when the speech was fearful, and weakest when the speech was comforting. However, this study also did not relate EEG responses to concurrent measures of temperament.

### The Current Experiment

In the current study, our primary goal again was to examine the relationship between parent-report measures of temperament and concurrent measures of frontal EEG asymmetry. While parental report measurements of temperament are useful in infant research, since temperament is hypothesized to have a biological basis, it is important to ascertain the relationship between parent-report measures of temperament and biological indices. Our secondary goal was to examine this relationship during emotionally salient conditions. Previous research has concentrated on measuring frontal EEG asymmetry during resting conditions. However, it is possible that eliciting frontal EEG asymmetry during motivationally-specific conditions may provide stronger results.

We thus examined associations between the subscales of the IBQ-R and frontal EEG asymmetry scores recorded during a combined series of neutral attentional and putatively emotional recording conditions in infants between 7 and 9 months of age. Emotional challenge tasks included both positively and negatively valenced vocal recordings [36], and films of snakes and elephants that have been shown in past research to differentially impact infant attention, presumably for affective reasons [37]. As in previous work, we predict that approach-related subscales of the IBQ-R (Approach, Duration of Orienting, Falling Reactivity, Perceptual Sensitivity, Smiling and Laughter, Soothability) will be related to greater left prefrontal asymmetry, while withdrawal-related subscales (Distress to Limitation, Fear) will be related to greater right prefrontal asymmetry.

## Methods

Our goal was to present infants with mildly positively and negatively valenced stimuli in order to assess individual differences in frontal EEG asymmetry. A corollary goal was to limit artifacts due to muscle, head or eye movements in EEG recordings, so instead of using behavioral paradigms known to induce major distress and crying, we used stimuli known in the developmental literature to induce milder positive and negative reactions. For example, previous research suggests that infants have a strong preference for non-threatening animals over a variety of other stimuli [38], [39]. Thus, animal films constitute a positive stimulus for infants. Further, recent research suggests that snakes may constitute a mildly negative stimulus for infants, as infants appear to associate snakes with fearful vocal stimuli [37], [39]. Thus, videos of snakes and non-threatening animals were selected as potential negative and positive visual stimuli, respectively. In addition to snake versus non-threatening animal videos, infants were presented with positive and negative vocal stimuli. These valenced vocal stimuli consisted of nonsense phrases that were either fearful (negative) or happy (positive) [36]. Visual and auditory stimuli were fully crossed in a 2×2 design, presented in randomized order. EEG data were collected throughout the presentation of all stimuli. Participants were highly attentive during these experimental conditions, as evidenced by the surprisingly low attrition rate for the experiment (only one infant was excluded).

### Participants

The participants were 23 7- to 9-month-old infants (*mean*: 8.0 mos, *range*: 6.8–9.1 mos; 11 females). The range of 7 to 9 months was chosen because it is the same range used in previous research discussed above examining associations between the IBQ and prefrontal processes in young infants (7 months [32]; 9 months [15], [33]). The sample was recruited from records of birth announcements in the local community and was predominantly Caucasian and middle-class. Parents of all participants provided informed consent. One infant was eliminated due to fussiness (and thus noisy EEG data), leaving a final sample of 22.

### Materials

#### Films

Video stimuli were six 12-sec color film clips from nature programs in which a snake (3 different snakes) slithered or a non-threatening animal (giraffe, rhinoceros, polar bear) walked at approximately the same slow rate across the screen.

#### Voice

We used six professionally-produced audio recordings of the same 2 nonsense phrases (“Hat sundig pron you venzy. Fee gott laish jonkill gosterr.”), spoken by 3 different men and 3 different women. Three of these recordings were spoken in a pleasant, happy-sounding tone of voice. The other three sounded distinctly frightened (These recordings have been scaled for emotional content and used in many studies of adult perception of emotion—cf., [36]).

#### Stimulus Presentation

Stimuli were projected onto a 91.4 cm by 121.9 cm white screen approximately 91 cm from the infant. The voices came from 2 speakers located on the sides of the screen. Each infant received 12 trials. All 6 films were presented twice, once accompanied by a fearful voice and once accompanied by a happy voice. Inter-trial intervals were 6 seconds. In full, stimuli were presented to each infant for a total of 204 seconds (approximately 3.4 minutes).

#### Temperament Measures

Upon entering the lab, parents were asked to fill out a modified version of the Infant Behavior Questionnaire – Revised (IBQ-R [31]). The IBQ-R is a revised version of the original IBQ [40]. The IBQ was revised in 2003 based on almost two decades of research using the IBQ [31]. The IBQ-R has a modified version of the original seven scales of the IBQ with nine additional scales. For more information, see Gartstein and Rothbart [31].

Because of the length of the questionnaire, only eight subscales of the IBQ-R were included in order to allow timely completion during their lab visit. The specific 8 scales were chosen specifically because of their relationship to approach and withdrawal tendencies (Rothbart, personal communication, April, 2004). These scales were Approach, Distress to Limitations, Duration of Orienting, Falling Reactivity, Fear, Smiling and Laughter, Perceptual Sensitivity, and Soothability. Brief descriptions of these temperament dimensions can be found in Table 1. The modified questionnaire took approximately 15 minutes for parents to complete.

**Table 1:** IBQ-R scale definitions from Garstein & Rothbart (2002).

Scale	Definition	Cronbach's alpha
Approach*	Approach, excitement and positive anticipation of pleasurable activities (12 items) *e.g. When given a new toy, how often did the baby get very excited about getting it?*	.70
Distress to Limitations‡	Fussing or crying when unable to perform a desired action (16 items) *e.g. When placed on his/her back, how often did the baby fuss or protest?*	.83
Duration of Orienting*	Duration of attention to, or interaction with, an object (12 items) *e.g. How often in the last week did the baby play with one toy or object for 5–10 min?*	.68
Falling Reactivity*	Rate of recovery from peak distress (13 items) *e.g. When going to bed at night, how often does your baby settle down to sleep easily?*	.60
Fear‡	Startle or distress to sudden stimulation (16 items) *e.g. How often in the last week did the baby startle to a sudden or loud noise?*	.89
Perceptual Sensitivity*	Detection of low intensity sensory stimuli (12 items) e.g. *How often does the infant look up from playing when the telephone rang?*	.90
Smiling and Laughter*	Frequency of smiling and laughing during caretaking or play (10 items) *e.g. How often during the last week did the baby laugh aloud in play?*	.61
Soothability*	Reduction in fussing or crying when caretaking is employed (18 items) *e.g. When rocking your baby, how often did s/he soothe immediately?*	.77

* = Putatively approach oriented, likely associated with left-lateralized prefrontal activity.

‡ = Putatively withdrawal oriented, likely associated with right-lateralized prefrontal activity.

#### Assessment of EEG

Concerns about working with small infants who may grow bored or fussy, and who may grab and damage EEG electrodes, lead to decisions to utilize a small number of electrodes, to limited recording times to as short as feasible, and to utilize the Cz reference (Heather Henderson, personal communication). These methodological decisions are discussed at greater length below. Tin electrodes in a stretch-lycra cap were used to record EEG at sites F3, F4, F7, F8, P3, and P4, from the international 10–20 system [41]. All sites were referenced online to Cz. The ground lead was on the midline just anterior to Fz. Electrode impedances were reduced to less than 5 kΩ following procedures outlined by Pivik and colleagues [42]. All sites were amplified by a factor of 20,000 with AC differential amplifiers (bandpass 0.1 and 300 Hz), and digitized continuously at 1000 Hz. Signal processing was conducted using Neuroscan's Edit software to complete the following analysis procedures (for review, see [43]). Prior to artifact screening, data files were filtered with a finite impulse response zero phase shift 161-point digital 60-Hz notch filter. Each file was visually screened for gross movement artifacts and for clipped signals; time periods containing such artifacts were removed from further analysis. Epochs with eye blinks were rejected manually as part of the gross movement screening. EEG data were event-coded according to recording condition (snake plus fear voice, snake plus happy voice, non-threatening animal plus fear voice, non-threatening animal plus happy voice), and concatenated within-condition to form four 36 s blocks of continuous data. From these blocks, EEG data were divided into 2-s epochs that overlapped by 1.5 s. The overlap of 75% was selected to compensate for the loss of data due to the imposition of a Hamming window prior to spectral analysis. A fast Fourier transform (FFT), using a Hamming window that tapered data at the distal 10% of each 2-s epoch (frequency resolution of 0.5 Hz), transformed data to power spectra, and the average power spectrum for each recording period was obtained. Total power within the alpha frequency band (6–9 Hz, see [44]) was extracted for each recording period, and these values were averaged across recording periods. Average alpha power values at each site were then log transformed using the natural log. A measure of EEG hemispheric asymmetry (right hemisphere compared to left hemisphere) was derived (ln[right] – ln[left]) for the mid-frontal (F4 and F3), lateral-frontal (F8 and F7) and parietal P4 and P3) regions. Because cortical alpha power is inversely correlated with cortical activity (see [43], for an extensive discussion; see also [45]), lower scores on this metric suggest relatively less left activity.

### Methodological considerations

As noted above, several methodological decisions were made in order to minimize infant fussiness while recording EEG data. These included utilizing a small number of electrodes, limiting the overall recording time, and utilizing the Cz reference. Indeed, our attrition rate was far lower than most similar studies; of 23 infants brought into the laboratory, only one became fussy enough to render EEG data unusable, a virtually unprecedented attrition rate, in this literature, of less than .5%. We believe our low attrition rate is attributable in part because our short and relatively undemanding EEG hookup, which allowed us to rapidly engage the infants in the experiment.

Our short recording time is contrary to a widely cited recommendation by Tomarken, Davidson, Wheeler, and Kinney [46] to record no less than 8 minutes of EEG for frontal EEG asymmetry scores of sufficient internal consistency reliability. However, Towers and Allen [47] have recently reported reasonably high internal consistency estimates of frontal EEG asymmetry (coefficient alphas of approximately .70) with as few as 40 epochs of two-second alpha power data. Recording times reported here within each recording state were 36 s in length (3×12 s for each video/voice combination). This length of EEG recording provides 51 epochs for use in our EEG analysis (36 s×3 epochs/2 s, subtracting the three epochs that cannot overlap). Of these 51 epochs, an average of 38 (approximately 75%) per recording condition were usable for the extraction of average alpha power. Average alpha power estimates from these epochs were extracted for each recording condition (snake plus fear voice, snake plus happy voice, non-threatening animal plus fear voice, non-threatening animal plus happy voice), and asymmetry scores were computed (see above). These asymmetry scores were then treated as four items in a scale reliability analysis for each of our regions of interest. This yielded internal consistency coefficient alphas of .95, .97 and .93 for the mid-frontal (F3–F4), lateral-frontal (F7–F8) and parietal (P3–P4) regions, respectively. When calculating reliability within recording conditions, the use of overlapping epochs may artificially inflate reliability estimates, because the EEG epochs are not strictly independent, even though the Hamming window procedure gives maximal weight to the center, non-overlapping portions of each EEG epoch [47]. In our approach, average alpha power estimates were first calculated for each recording condition separately, and these averages were used to estimate reliability, thus minimizing the non-independence problem in reliability estimation. Ultimately, our estimates of reliability are highly comparable to those reported elsewhere in this literature (cf., [43]).

In addition to the small number of electrodes and the relatively short recording times, the Cz reference has been deemed problematic in frontal EEG asymmetry research [43], [48], [49]. Nevertheless approximately 80% of infant frontal EEG asymmetry studies recently reviewed by Coan and Allen [12] have utilized the Cz reference, likely for reasons similar to our own. Moreover, Coan et al. [20] recently proposed that variance due to reference scheme was negligible when emotional challenges were used to elicit individual differences in frontal EEG asymmetry. Indeed, Coan and colleagues [20] observed that the average correlation between the Cz reference and other references (e.g., average and linked mastoids) was *r* = .63 when affective stimuli were used, in contrast to an average correlation of *r* = .18 at rest.

Ultimately, these past observations, coupled with the very high internal consistency estimates we have observed, suggest that the quality of our EEG data did not suffer greatly as a result of our attempts to minimize the fussiness of our infant participants by selecting a subset of electrodes and recording for small periods of time. We note again that our attempt to minimize infant fussiness and attrition was highly successful.

### Procedure

Upon entering the lab, each infant was fitted with a stretch lycra EEG cap described above. Fitting and preparing the cap took approximately 15 minutes. The infant was seated on a parent's lap in front of the screen. Stimuli were presented using DMDX presentation software [50]. The experimenter manually began the DMDX presentation once the infant's eyes were situated on the screen. From this point, the presentation of trials was controlled automatically by the DMDX program, which presented the 12 trials in randomized order to each participant. In between trials, infant attention was maintained by a 6 s blinking green dot that appeared in the center of the screen accompanied by a “dinging” sound. The next trial commenced automatically unless the experimenter observed the infant's attention wandering, in which case it was possible to replay the blinking green dot until the infant's attention returned to the screen. The entire procedure lasted approximately 30 minutes.

### Data analysis

Linear mixed models (SPSS) were conducted separately for each temperament measure. The models included main effects for EEG asymmetry scores at F34 (M = .78, SD = .26), F78 (M = .70, SD = .27) and P34 (M = .80, SD = .25), animal (snake versus non-threatening) and voice (fearful versus happy). All effects were specified as fixed. Models were calculated using a diagonal repeated covariance structure, and utilizing a type 1 sum of squares to deal with colinearity among asymmetry scores, which was high with all correlation coefficients between asymmetry scores greater than or equal to *r* = .80. Parietal asymmetry scores were included first in order to 1) allow for our comparison region to have first priority in predicting temperament scores and 2) test for prefrontal effects in predicting temperament scores after first adjusting for variance that prefrontal sites may share with the parietal region. Also included were interaction effects between animal and voice, as well as interactions between EEG asymmetry scores and animal and voice valence conditions. Following the identification of main and interaction effects, effects were decomposed using simple regressions per Aiken and West [51].

## Results

### IBQ-R Intercorrelations

The inter-item alpha coefficients for IBQ-R subscales ranged from .60 to .90 and are listed in Table 1. Table 2 presents intercorrelations among the six dimensions of temperament assessed by the IBQ-R subscales reported here. Significant positive associations were observed between Distress to Limitations and Fear (*r* = .48, *p*<.05); and Fear and Perceptual Sensitivity (*r* = .54, *p*<.05). Significant negative associations were observed between Fear and Duration of Orienting (*r* = −.43, *p*<.05); Falling Reactivity and Approach (*r* = −.47, *p*<.05); and Falling Reactivity and Distress to Limitations (*r* = −.51, *p*<.05).

**Table 2:** Intercorrelations between the dimensions of the IBQ-R scale.

	Distress to Limitations	Fear	Duration of Orienting	Smiling and Laughter	Soothability	Falling Reactivity	Perceptual Sensitivity
**Fear**	.48*						
**Duration of Orienting**	−.25	−.43*					
**Smiling and Laughter**	.26	.39	−.04				
**Soothability**	−.14	−.08	.06	.22			
**Falling Reactivity**	−.51*	−.20	−.01	−.19	.12		
**Perceptual Sensitivity**	.27	.54*	−.17	.16	−.01	−.08	
**Approach**	.39	−.07	.14	.14	.06	−.47*	−.09

* = p<.05.

### EEG and Experimental conditions

Linear mixed models—one for each cortical region—were used to assess possible state effects of experimental condition on EEG asymmetry at any region. No effects of animal type (snake versus non-threatening) voice (fearful versus happy), or their interaction, were observed. All variables were subjected to maximum normed residual tests [52] for possible outliers.

### EEG and IBQ-R

#### Prefrontal Regions

**Figure 1:**
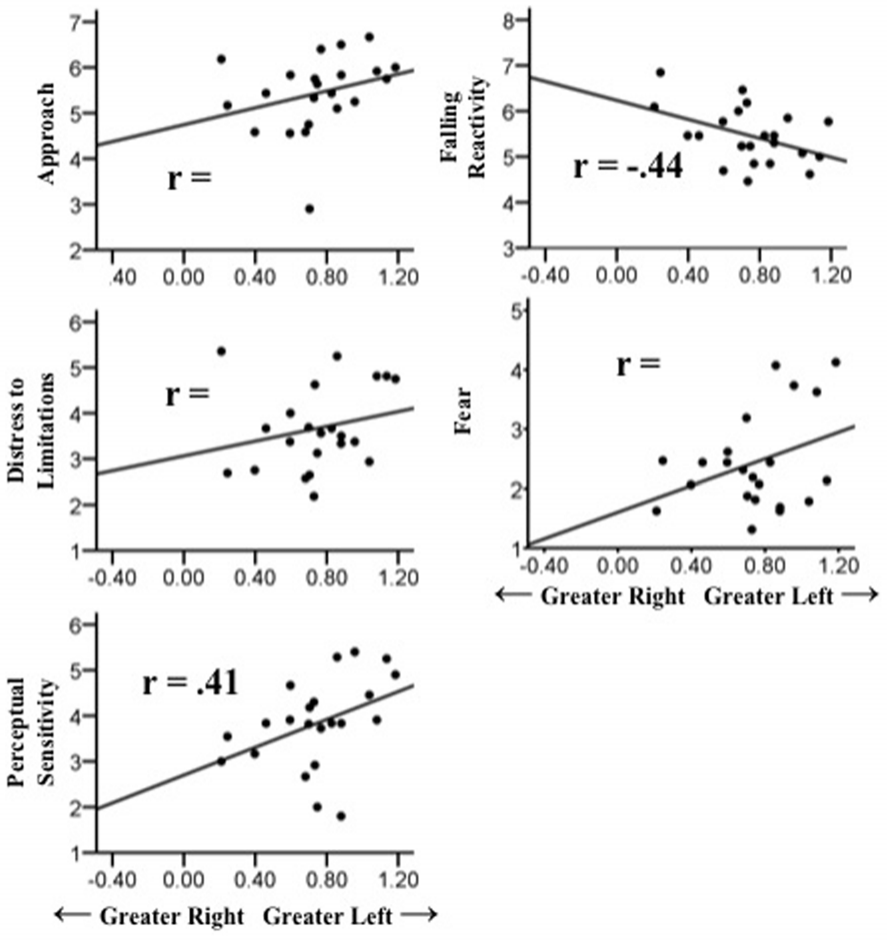
Scatterplots of correlations between mid-frontal (F3–F4) EEG asymmetry and the Approach, Distress to Limitations, Falling Reactivity, Fear and Perceptual Sensitivity scales of the IBQ-R.

**Figure 2:**
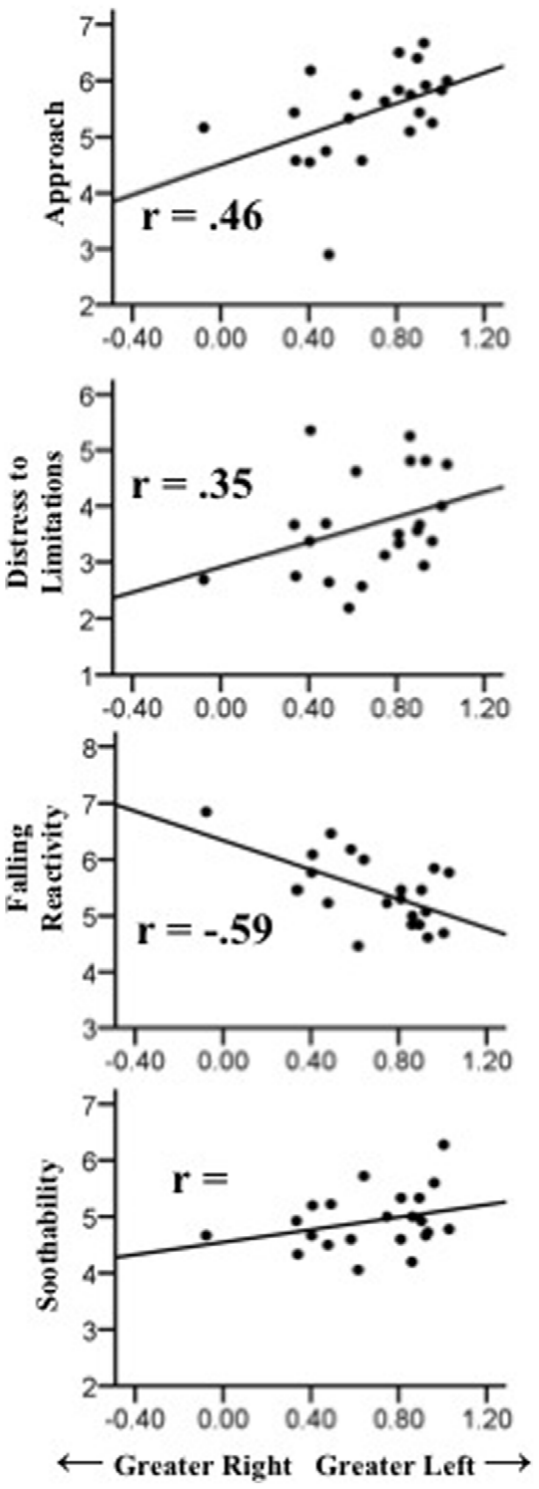
Scatterplots of correlations between lateral-frontal (F7–F8) EEG asymmetry and the Approach, Distress to Limitations, Falling Reactivity, and Soothability scales of the IBQ-R.

Linear mixed models were used to assess 1) the effects of animal (snake versus non-snake) and valence (happy versus fearful) on EEG asymmetry, and 2) the degree to which EEG asymmetry predicted infant temperament, either independently or as a function of animal and valence conditions. Infant EEG asymmetries were robustly related to parental reports of infant temperament, regardless of experimental condition. Table 3 details significant main effects of asymmetries over the frontal regions on IBQ temperament measures. Over the mid-frontal region, EEG asymmetry significantly predicted Approach, *F*(1, 201) = 14.27, *p*<.001, *r* = .29; Distress to Limitations, *F*(1, 196) = 7.40, *p*<.01, *r* = .23; Fear, *F*(1, 200) = 17.65, *p*<.001, *r* = .36; Perceptual Sensitivity, *F*(1, 202) = 30.84, *p*<.001, *r* = .41; Falling Reactivity, *F*(1, 202) = 49.21, *p*<.001, *r* = −.44. In other words, Approach, Distress to Limitations, Fear, and Perceptual Sensitivity were associated with greater left frontal activation, and Falling Reactivity was associated with greater right frontal activation. Similar findings were obtained over the lateral-frontal region, with EEG asymmetries predicting Approach, *F*(1, 165) = 20.76, *p*<.001, *r* = .46; Distress to Limitations, *F*(1, 169) = 14.18, *p*<.001, *r* = .35; Soothability, *F*(1, 166) = 23.17, *p*<.001, *r* = .30; and Falling Reactivity, *F*(1, 179) = 39.75, *p*<.001, *r* = −.59, with Approach, Distress to Limitations, and Soothability significantly related to left frontal activation, and Falling Reactivity significantly related to right frontal activity (see Figures 1 and 2).

**Table 3:** Significant Effects of Linear Model Models designed to predict infant temperament using mid-frontal, lateral-frontal and parietal EEG asymmetry.

Region	Source	df	*F*	*p*	*r*
Mid-frontal					
	Approach	1, 225	14.27	<.001	.29
	Distress to Limitations	1, 225	7.55	<.01	.23
	Falling Reactivity	1, 228	49.86	<.001	−.44
	Fear	1, 227	18.65	<.001	.36
	Perceptual Sensitivity	1, 227	32.46	<.001	.41
Lateral-frontal					
	Approach	1, 191	20.58	<.001	.46
	Distress to Limitations	1, 193	14.50	<.001	.35
	Falling Reactivity	1, 204	36.84	<.001	−.59
	Soothability	1, 192	23.95	<.001	.30

N = 22; Note that denominator degrees of freedom are estimated from Satterthwaite approximations without exact F distributions.

Degrees of freedom were estimated for the population based on a restricted maximum likelihood procedure, and were rounded to the nearest whole number. Over the mid-frontal region, higher EEG asymmetry scores were related to higher Approach, *F*(1, 201) = 14.27, *p*<.001, *r* = .29; Distress to Limitations, *F*(1, 196) = 7.40, *p*<.01, *r* = .23; Fear, *F*(1, 200) = 17.65, *p*<.001, *r* = .36; Perceptual Sensitivity, *F*(1, 202) = 30.84, *p*<.001, *r* = .41; and lower Falling Reactivity, *F*(1, 202) = 49.21, *p*<.001, *r* = −.44;. Similarly, over the lateral-frontal region, higher EEG asymmetries were related to higher Approach, *F*(1, 165) = 20.76, *p*<.001, *r* = .46; Distress to Limitations, *F*(1, 169) = 14.18, *p*<.001, *r* = .35; Soothability, *F*(1, 166) = 23.17, *p*<.001, *r* = .30; and lower Falling Reactivity, *F*(1, 179) = 39.75, *p*<.001, *r* = −.59.

It is important to note that the Approach scale was discovered to contain an outlier. Removal of this outlier resulted in no change in the overall pattern of results in either the mid-frontal region, *F*(1, 181) = 17.13, *p*<.001, *r* = .37, or the lateral-frontal region, *F*(1, 149) = 20.30, *p*<.001, *r* = .52.

#### Parietal Region

Unexpectedly, some associations between EEG asymmetry over the parietal region and IBQ-R measures were observed, although these associations were relatively infrequent or dependent upon voice condition. For example, parietal EEG asymmetry did predict Distress to Limitations, *F*(1, 169) = 10.01, *p*<.01, *r* = .38, much as the prefrontal regions did. Falling Reactivity was also predicted by parietal asymmetry, *F*(1, 193) = 4.24, *p*<.05, *r* = .35, although this association appeared to be dependent upon valence, as revealed by a significant parietal asymmetry by valence interaction, *F*(1, 196) = 5.96, *p*<.02. Decomposition of this interaction revealed that parietal EEG asymmetry was negatively associated with Falling Reactivity during positive valence voice conditions, *β* = −.38, but unrelated to Falling Reactivity during negative voice conditions, *β* = −.05.

## Discussion

The objective of the current research was to examine associations between frontal EEG asymmetry—a putative measure of temperament and endophenotypic marker of risk for and resilience from affective disorders—and parental reports of early infant temperament. Results suggest that frontal EEG asymmetry is robustly related to many of the subscales of the IBQ-R. However, our results contradict some—certainly not all—previously reported associations between frontal EEG asymmetry and infant temperament. Contradictions between our findings and previously reported associations between frontal EEG asymmetry and temperament reported in the past could be due to many things, not least methodological differences between our work and past work in this area.

Our methodology strongly emphasized minimizing participant attrition rates, which have commonly been quite high in past research of this type [15], [53], [54], even though doing so introduced other methodological constraints (e.g., low number of electrode sites, dependence on the Cz reference scheme) that must be considered in interpreting our results. For example, in our study, a large number of children who might have been excluded from past studies due to fussiness were able to complete our experiment. Second, our sample of infants was slightly younger (as young as 7 months old) than those reported in previous studies. Third, past researchers have often utilized a strategy of selecting or classifying infants who were more strongly left or right lateralized (e.g., [15], [33]), whereas our sample was more uniformly left-lateralized throughout our experiment, and we treated frontal EEG asymmetry as a more continuous measure. Indeed, with this last point in mind, it could be that we have observed continuous associations between frontal EEG asymmetry and IBQ measures of temperament among infants who are more generally left frontal, or who were put into a predominantly left frontal state during our experimental paradigm. Close scrutiny of our internal consistency estimates and the scatterplots of associations we identified suggest, in any case, that whatever the ultimate source of our contradictory results, they are unlikely to be due to chance alone. In the following discussion, we consider other possible explanations for our observations.

### Frontal EEG Asymmetry and the Infant Behavior Questionnaire

Several of the associations observed here between frontal EEG asymmetry and IBQ-R measures conceptually mirror a vast and growing database of theoretical and empirical contributions linking frontal EEG asymmetry to personality, emotion, and emotion-regulation, as well as both risk for and resiliency against affective disorders in the face of stressful life situations [12], [17], [25], [26], [55]. For example, left-lateralized asymmetry scores were associated with higher parental ratings of approach- or engagement- related constructs such as Approach and Soothability. However, it merits repeating that virtually all observed statistical associations were characterized by a pattern of left-lateralized prefrontal activity corresponding with higher IBQ-R scores. We expected a pattern of relatively greater *right* prefrontal activity to correspond with higher fear scores, and confusingly observed precisely the opposite. Moreover, scatterplots (Figures 1 and 2) of all observed associations, in addition to maximum normed residual tests on all variables, suggest these associations were due neither to artifacts of measurement error nor to outliers in our data.

### Fear, Perceptual Sensitivity, Limitation Distress and Falling Reactivity

Ultimately, the positive associations we observed between frontal EEG asymmetry and many of the putatively withdrawal-oriented subscales of the IBQ-R may indicate that under some circumstances, those scales are capable of tracking approach-related variance in early infant temperament. Certainly, associations between relatively greater left frontal EEG asymmetry and negative affect are not unprecedented, and it is possible that the conditions under which our EEG recordings were obtained in this study selected primarily for individual differences in approach-oriented capabilities in brain activity (cf., [20]), even if the valence associated with those capabilities was negative or at the very least ambiguous. For example, studies of *anxious apprehension* (which might also be characterized as hypervigilance) have associated negative affective states with left lateralized prefrontal activity [56], [57], although this work has emphasized the effect of ruminative language processing in Broca's Area as an explanation of left-lateralization instead of some sort of negatively valenced state of approach motivation. Although ruminative language processing is unlikely to account for observations of left-lateralized frontal EEG asymmetry in infants, it is still possible that an apprehension-like or vigilance response, perhaps developing pre-verbally even in young infants, may involve the “interpreter system” of the left prefrontal cortex—a system thought to underlie information integration and hypothesis generation as the brain attempts to accurately predict important outcomes (e.g., [58], [59]).

The existence of negative associations between Distress to Limitations and Falling Reactivity, the latter of which is actually an inverse measure of how long it takes for an infant to recover affectively after falling down, suggests a general capacity for frustration that may have been captured by our frontal EEG asymmetry measure. A substantial literature links relatively greater left prefrontal activity to anger and frustration in adolescents and adults [22]; and anger is a fundamentally approach-oriented emotion [60]. That is, it functions to compel individuals to actively engage their environment in the face of unmet or blocked needs and resources.

A potentially simpler explanation for the positive associations between pFA and the withdrawal-oriented subscales of the IBQ-R is that the stimuli used in the current experiment induced a state of quiet attentiveness in the infant participants. If this is indeed the case, the current findings suggest that individual differences in the state of quiet attentiveness can be captured in the IBQ-R. Future research may be able to address this possibility more directly.

### Approach and Soothability

Evidence suggests that positive affect—by virtue of its generally approach-oriented motivational base—is associated with relatively greater left prefrontal activity [27]. In the IBQ-R, the Approach scale is intended to measure tendencies toward excitement at the possibility of pleasurable activities. Beyond simply pleasurable anticipation, however, Davidson [13], [21] has pointed to a pattern of increased left frontal EEG asymmetry as consistently associated with better mental health outcomes, possibly by virtue of its association with better self-regulatory capabilities [see also 61].

Of course, infants are relatively poor self-regulators. Indeed, at 6 to 9 months of age, infants are still highly dependent upon their caregivers for soothing and other regulatory support. This is a form of *co-regulation* [62], [63], a process by which one individual regulates the emotional and physiological responding of the other. In infants, what begins as regulation by the caregiver of the infants' physiological needs *via* the infant's expressed affect gradually becomes the regulation of the infant's affect, per se [62]. Associations between relatively greater left prefrontal activity and higher soothability scores suggest the possibility that infants high in soothability are more receptive to emotional regulation in the mode most appropriate to their age—which is soothing by their caregiver.

### Methodological considerations and limitations

As mentioned above, most previous research examining the links between frontal EEG asymmetry and temperament have measured EEG during emotionally neutral or resting conditions. However, Coan et al. [20] have argued that such strategies may not capture all prefrontal processes involved in directing or regulating emotional responses. In general, the frontal EEG asymmetry literature has produced several instances of inconsistent or contradictory results [12], the full extent of which may be unknown due to the “file drawer” problem [64]. Following the recommendations of Coan et al. [20], we chose to record EEG during emotionally challenging stimuli. We intended to draw out avoidance-related patterns of prefrontal activity using both fearful voices and images of snakes during EEG recording, but it may be either that our stimuli were not sufficiently challenging or that our stimuli inadvertently did just the opposite, and drew out approach-related patterns of prefrontal activity instead. Observational evidence of common notions of avoidance-oriented fear behavior is inconsistent in infants until around 6–8 months of age [65], just about the age of the infants observed in this research. Although we did not see the avoidance-based results we expected, correlations reported here conceptually add to evidence presented by Coan et al. [20] that frontal EEG asymmetries recorded during emotional challenges may differ markedly from those recorded at rest, possibly increasing the sensitivity of the frontal EEG asymmetry score generally, but also altering the direction of associations with criterion measures.

This use of emotional challenges may also have increased sensitivity to effects extending to the parietal cortex. As noted above, these associations were infrequent or dependent upon the voice condition, but in general, their direction was in line with prefrontal effects. Moreover parietal asymmetry scores were highly correlated with frontal asymmetry scores, an observation that is contrary to many similar observations in older child or adult samples. On the one hand, the simplest explanation may be that the magnitude of the parietal/frontal correlations reported here are a function of the proximity of parietal leads to frontal leads in our small infant sample (relative to older samples), in conjunction with the increased frontal activity resulting from the emotional challenges in our design (cf., [20]). EEG signals are often fairly diffuse, rendering spatial specificity somewhat difficult [43]. If parietal effects partially reflect the diffusion of frontal effects across proximally placed scalp electrodes, we might expect the pattern of parietal effects we actually did observe—effects that appear similar to frontal effects, although weaker and less consistent. On the other hand, parietal EEG asymmetries associated emotional responding and temperament are themselves not unprecedented. For example, Davidson, Schaffer, and Saron [66] observed a pattern of relatively greater left parietal activity among individuals suffering from depression. Schmidt & Fox [67] observed that individuals low in shyness and high in sociability displayed relatively greater right parietal activity, while individuals low in shyness and low in sociability displayed relatively greater left parietal activity. Heller and Nitschke [68] have proposed that certain forms of anxiety should correspond with relatively greater right parietal activity. Although asymmetries over the parietal cortex associated with emotional responding have been observed for many years, models of asymmetrical parietal activity and emotion are elusive, especially in comparison to asymmetrical prefrontal activity.

The entire distribution of infants in this study showed evidence of being relatively more left prefrontally active than right. On the one hand, this may constitute further evidence that our recording conditions inadvertently selected for approach-related prefrontal activity, with individuals distributed non-randomly along an axis of that activity. On the other hand, this seemingly peculiar distribution is itself not without precedent. Marshall and Fox [69] reported that in their own work, they have often observed that their research participants—particularly infants—show a pattern of predominantly left prefrontal activity in the laboratory. Because frontal EEG asymmetry scores have a theoretical mid-point indicating perfect symmetry between hemispheres, it is perhaps natural to expect such symmetry to correspond with the arithmetic mean of most distributions, but this need not be the case. In fact, at least one prominent theory of affect suggests it should not be—that in fact individuals are most likely to show relatively greater left prefrontal activity (indicating an approach orientation), most of the time. This would reflect the *positivity offset*, whereby the baseline state for most individuals is mildly positive and approach-oriented, which facilitates exploration and interaction with the environment [70].

In any case, it remains true that a limitation of the current work may be that our emotional challenges were either not constructed correctly, or were not powerful enough, to elicit individual differences in withdrawal-oriented neural responding. Research with older infants has used experimental conditions such as stranger approach or maternal separation to elicit negative affect (i.e., [53], [71]). It is possible that stronger negative experimental conditions may have more clearly elicited individual differences in withdrawal-related response capabilities. An additional limitation of this work is that our emotionally challenging conditions were not compared directly with a neutral or baseline condition. It is possible that interesting differences would be found if emotional stimuli were compared to neutral stimuli. In future research, challenging emotional conditions should be compared with baseline or resting conditions.

### Summary and Conclusion

In conclusion, the current research demonstrates a consistent pattern of relatively greater left prefrontal activity corresponding with higher scores on many subscales of the IBQ-R, suggesting that frontal EEG asymmetry is a reliable biological correlate of parent-rated temperament even in early infancy. Importantly, these observations suggest that many of the scales of IBQ-R are capable of tracking variance in approach-related patterns of frontal EEG asymmetry, even and perhaps especially in infants younger than 9 months of age, where withdrawal or avoidance related behavior is still developing and may be inconsistently measurable. This study differs from previous work in that individual differences in prefrontal activity were recording during experimentally manipulated emotional challenges, as opposed to neutral or resting conditions. This may account for the number of associations we observed between frontal EEG asymmetry and the IBQ-R scales, which were numerous and large in comparison to past research in this area. Although we were surprised to find that higher scores on the IBQ-R Fear scale corresponded with relatively greater left prefrontal activity, similar associations with other scales of the IBQ-R (e.g., the Approach scale) correspond well with past research, and the possibility that the IBQ-R Fear scale is capable of tracking approach-related variance among more strongly left-frontally active individuals is an idea worth exploring in future research. Moreover, it may be possible that our experimental paradigm engaged a pre-verbal form of anxious apprehension in some of our infants that may itself account for our positive association between frontal EEG asymmetry and several of the IBQ's putatively withdrawal-oriented subscales. Ultimately, this study provides an important new look at associations between prefrontal asymmetries putatively related to infant temperament and the most commonly used parental report instrument for measuring infant temperament, the IBQ-R. We look forward to continued work in this area.
